# Treg-derived IFN-γ supports the differentiation of Th1-Treg in tumor immunity and autoimmunity

**DOI:** 10.3389/fimmu.2026.1756194

**Published:** 2026-05-12

**Authors:** Ayumi Kuratani, Masaaki Okamoto, Sekar Arumsari, Takashi Kobayashi, Miwa Sasai, Masahiro Yamamoto

**Affiliations:** 1 Department of Immunoparasitology, Research Institute for Microbial Diseases, Osaka University, Suita, Osaka, Japan; 2 Laboratory of Immunoparasitology, World Premier International Immunology Frontier Research Center, Osaka University, Suita, Osaka, Japan; 3 Department of Infectious Disease Control, Faculty of Medicine, Oita University, Oita, Japan; 4 Division of Pathophysiology, Research Center for GLOBAL and LOCAL Infectious Diseases, Oita University, Oita, Japan; 5 Department of Immunoparasitology, Center for Infectious Disease Education and Research, Osaka University, Suita, Osaka, Japan

**Keywords:** EAE, IFN-γ, platelet factor 4 (PF4), Th1-Treg, TME, Treg

## Abstract

Regulatory T cells (Tregs) within the tumor microenvironment (TME) exhibit functional heterogeneity, including a Foxp3^+^T-bet^+^ subset known as Th1-type Treg (Th1-Treg) cells that exert potent immunosuppressive activity. Although IFN-γ signaling is essential for Th1-Treg differentiation, the cellular source of IFN-γ in tumors has remained unclear. Here, we reveal that Treg cells themselves are one source of IFN-γ, which enhances the Th1-Treg induction. Treg-derived IFN-γ acts in an autocrine manner to stabilize T-bet expression and maintain the Th1-Treg phenotype, while Arg1^+^ tumor-associated macrophage (TAM)–derived platelet factor 4 (PF4) amplifies this loop by inducing *Ifng* transcription in Tregs. Conditional deletion of *Ifng* in Foxp3^+^ cells impaired Th1-Treg differentiation both in tumors and in the spleen, indicating that Treg-derived IFN-γ contributes to Th1-Treg maintenance at local and systemic levels. Moreover, Treg-derived IFN-γ similarly promoted Th1-Treg generation during experimental autoimmune encephalomyelitis, suggesting its role in type 1 inflammatory environments. Together, these findings reveal that Treg-derived IFN-γ contributes to a positive feedback circuit, acting in concert with other IFN-γ sources and TAM-derived PF4 to sustain Th1-Treg differentiation and accumulation in tumors, thereby reinforcing immunosuppression.

## Introduction

Regulatory T (Treg) cells are essential for maintaining immune homeostasis by suppressing excessive immune activation and preventing autoimmunity (1, 2). However, in pathological settings such as cancer, their potent immunosuppressive activity can inhibit effective antitumor immune responses. Within tumors, Treg cells suppress effector T cell activation, thereby promoting immune evasion and tumor progression (3–5).

Recent studies have revealed that Treg cells are not a homogeneous population but comprise functionally distinct subsets that adapt to local immune environments (6). Foxp3 serves as the lineage-defining transcription factor of Treg cells (7, 8), whereas they can also co-express the Th1-lineage transcription factor T-bet encoded by Tbx21 gene (9, 10). This subset, known as Th1-type Treg (Th1-Treg) cells, is characterized by high expression of CXCR3 and exhibits enhanced suppressive activity, particularly in the context of type 1 immune responses (11–14). These T-bet^+^ Treg cells have been shown to suppress Th1 and CD8^+^ T-cell responses during infection and tumor progression.

Th1-Treg cells preferentially accumulate within the tumor microenvironment (TME) relative to non-tumor tissues (15, 16), suggesting that TME selectively promote their differentiation and maintenance. Previous studies have begun to clarify how Th1-Treg cells accumulate within the TME. We recently reported that Arg1^+^ TAMs secrete platelet factor 4 (PF4), which polarizes Treg cells into Th1-Treg cells in a CXCR3-dependent manner (17). The activity of PF4 was highly dependent on IFN-γ receptor signaling in Treg cells, suggesting that IFN-γ acts as a critical cofactor for Th1-Treg differentiation. IFN-γ, however, is classically produced by CD8^+^ T cells, NK cells, and Th1 effector cells, where it mediates antitumor immunity (18). Paradoxically, several studies have also shown that IFN-γ signaling drives the acquisition of a Th1-like phenotype in Treg cells *in vitro* (12, 19, 20). Indeed, recent *in vivo* studies have demonstrated that IFN-γ promotes the generation of Th1-like Tregs not only within tumors but also in tumor-draining lymph nodes, where these cells suppress dendritic cell function and limit cytotoxic T-cell priming (21). Together, these findings indicate that IFN-γ plays an important role in Th1-Treg differentiation, yet the precise source of IFN-γ within tumors remains unclear. Whether the IFN-γ that promotes Th1-Treg differentiation originates from conventional effector lymphocytes, from Treg cells themselves, or from both has not been clearly defined. In this study, we aimed to identify the cellular sources of IFN-γ that support Th1-Treg differentiation, and to elucidate how PF4 secreted by Arg1^+^ TAMs reinforces this process within the tumor microenvironment.

## Results

### IFN-γ is required for Th1-Treg differentiation in tumors

**Figure 1:**
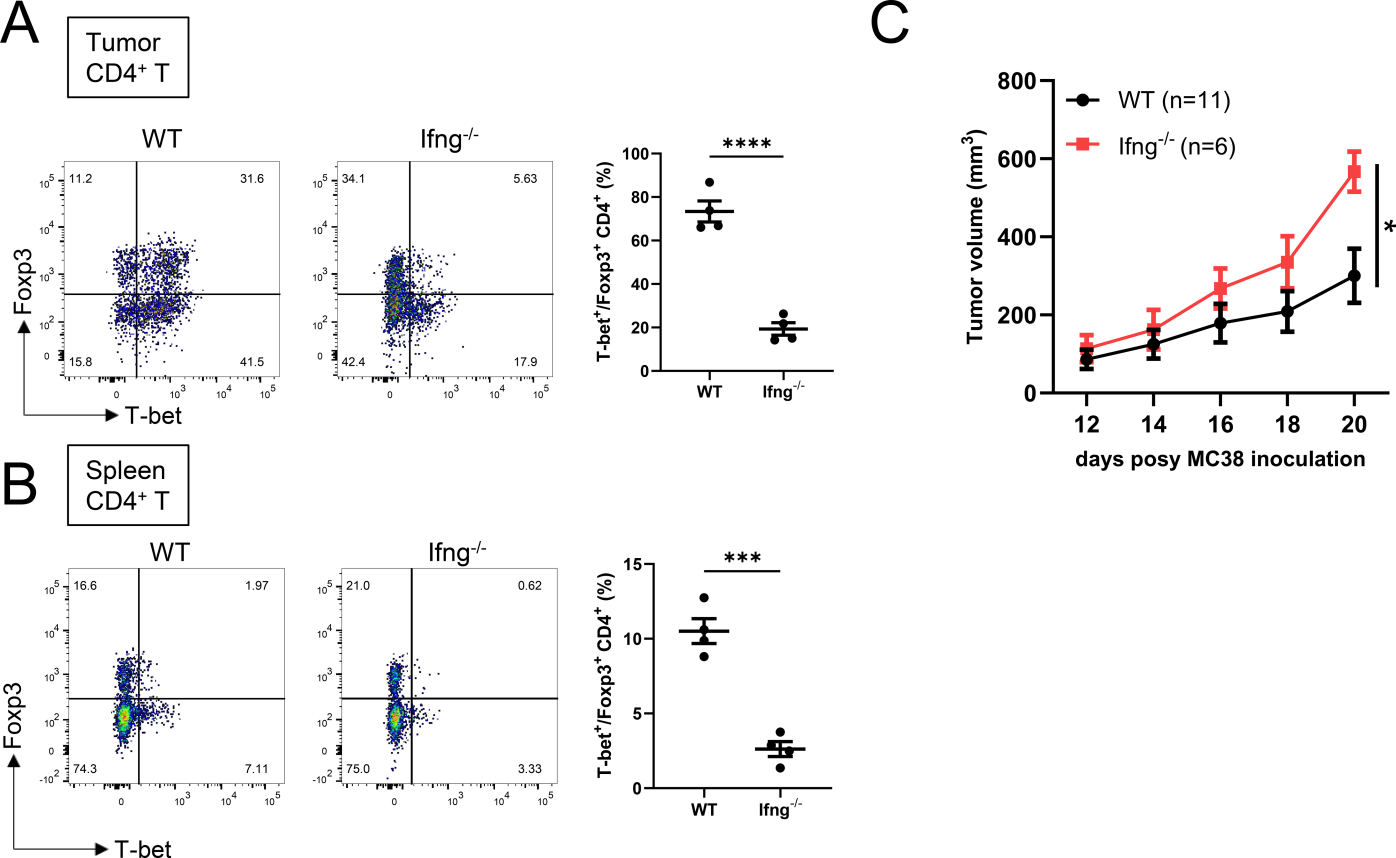
IFN-γ is required for Th1-Treg differentiation in tumors. **(A, B)** Flow cytometry analysis of CD4^+^ T cells derived from tumors **(A)** or spleens **(B)** of WT or Ifng^−^/^−^ mice subcutaneously implanted with MC38 cells. Left, representative FACS plots; right, frequencies of T-bet^+^ cells among Foxp3^+^CD4^+^ T cells (n = 4 mice per group). **(C)** Growth of s.c. implanted MC38 tumors in WT or Ifng^−^/^−^ mice (indicated numbers of mice per group). Data are mean ± SEM and pooled from two to three independent experiments. Statistical analysis was performed using two-tailed Student’s t-tests. *P < 0.05; ***P < 0.001; ****P < 0.0001.

To assess the role of IFN-γ in the generation of Th1-Treg cells, we first analyzed tumor-bearing Ifng^−^/^−^ mice. Flow cytometric analysis revealed a marked reduction in both the frequency and absolute number of Foxp3^+^T-bet^+^ CD4^+^ T cells (Th1-Tregs) in tumors compared with wild-type (WT) controls (**Figures 1A, B**). A similar decrease in the frequency of Th1-Tregs was also observed in the spleens of Ifng^-/-^ mice, although the absolute number of these cells in the spleen was comparable between the two groups (**Supplementary Figure 1**). These findings indicate that IFN-γ signaling supports Th1-Treg differentiation both within tumors and in peripheral lymphoid tissues. Regarding tumor progression, Ifng^−^/^−^ mice exhibited significantly accelerated tumor growth (**Figure 1C**), consistent with the well-established anti-tumor functions of IFN-γ. Notably, IFN-γ deficiency also led to impaired Th1-Treg differentiation, a highly immunosuppressive T cell subset.

**Figure 2:**
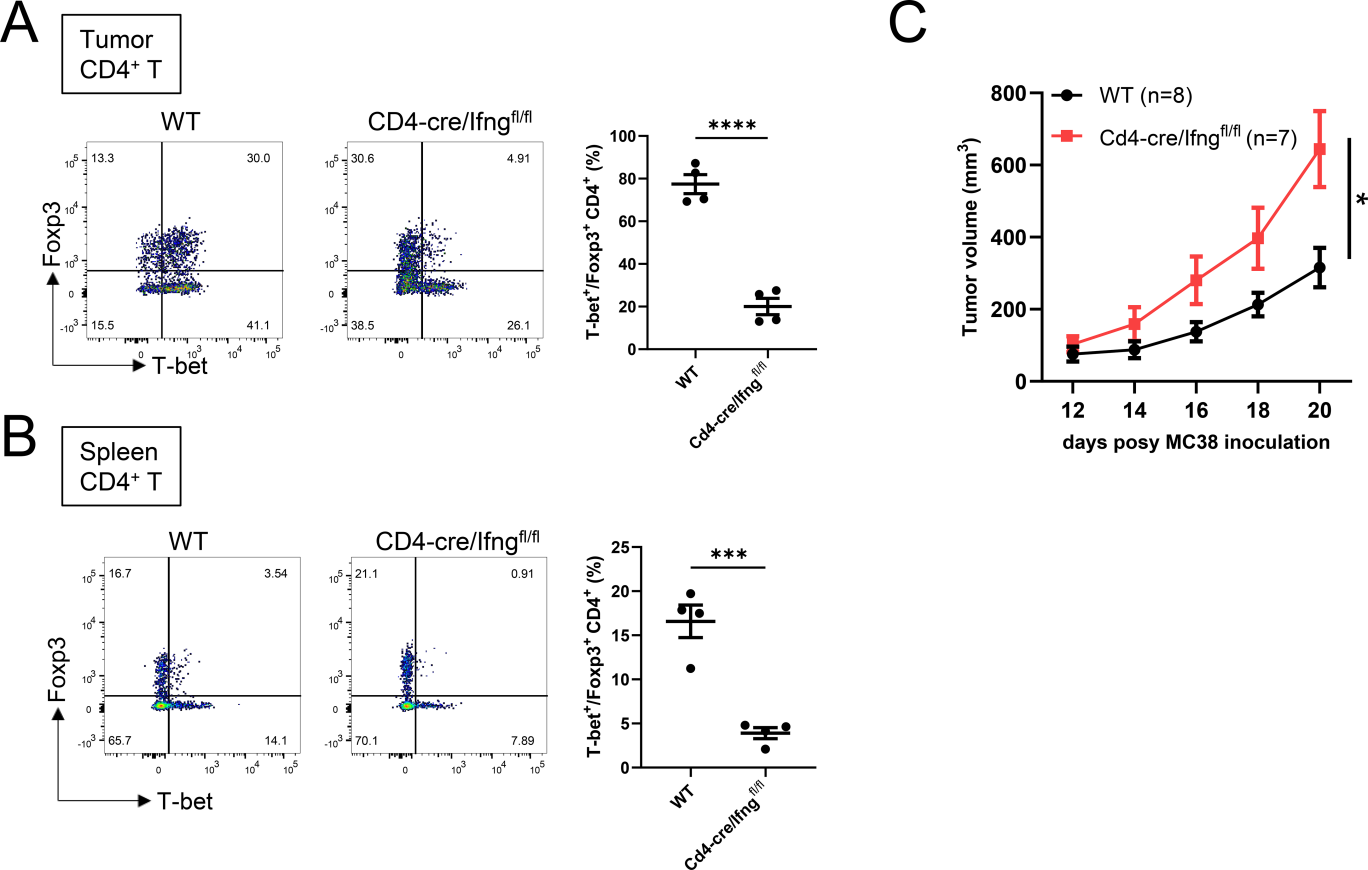
T cell–derived IFN-γ is required for Th1-Treg differentiation in tumors. **(A, B)** Flow cytometry analysis of CD4^+^ T cells derived from tumors **(A)** or spleens **(B)** of WT or CD4-Cre/Ifng^fl/fl^ mice subcutaneously implanted with MC38 cells. Left, representative FACS plots; right, frequencies of T-bet^+^ cells among Foxp3^+^CD4^+^ T cells (n = 4 mice per group). **(C)** Growth of s.c. implanted MC38 tumors in WT or CD4-Cre/Ifng^fl/fl^ mice (indicated numbers of mice per group). Data are mean ± SEM and pooled from two to three independent experiments. Statistical analysis was performed using two-tailed Student’s t-tests. *P < 0.05; ***P < 0.001; ****P < 0.0001.

To determine the cellular source of IFN-γ relevant for Th1-Treg differentiation, we generated T cell–specific *Ifng* conditional knockout (CD4-Cre/Ifng^fl/fl^) mice. Flow cytometric analysis revealed that CD4-Cre/Ifng^fl/fl^ mice exhibited markedly decreased frequencies and absolute numbers of Th1-Treg cells in both tumors and spleens compared with control mice (**Figures 2A, B**, and **S2**). We further found that tumor growth was significantly accelerated in CD4-Cre/Ifng^fl/fl^ mice (**Figure 2C**), consistent with the phenotype observed in global Ifng^-/-^ mice. These results suggest that T cell-derived IFN-γ is the main driver of Th1-Treg differentiation and demonstrate that IFN-γ is a key regulator of both antitumor effector responses and immunoregulatory T cell programs.

### Treg cells themselves produce IFN-γ

**Figure 3:**
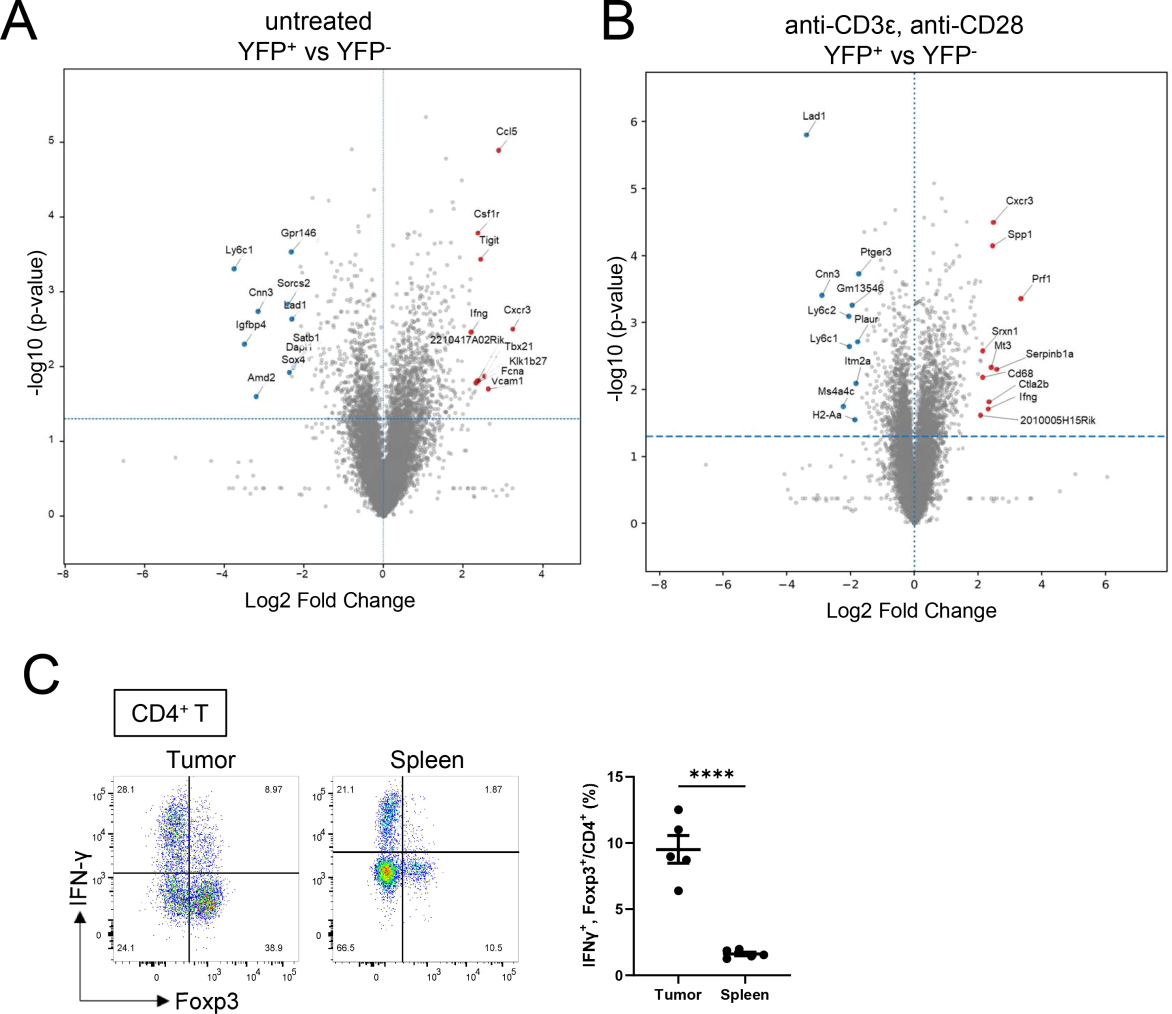
Treg cells themselves produce IFN-γ **(A, B)** Volcano plots based on RNA-seq analysis of YFP^+^ and YFP^−^ Treg cells isolated from the spleens of Foxp3-Cre/Tbx21-Flp/VeDTR(LF) mice. RNA-seq data were reanalyzed from a previously published dataset (16). **(A)** Comparison of unstimulated cells. **(B)** Comparison of cells stimulated *in vitro* with anti-CD3 and anti-CD28 antibodies in the presence of IL-2 for 3 days. Selected genes are highlighted. **(C)** Flow cytometry analysis of Foxp3 and IFN-γ expression in CD4^+^ T cells isolated from tumors or spleens of MC38-bearing WT mice and stimulated *ex vivo* with PMA and ionomycin. Representative FACS plots are shown. Data are mean ± SEM and pooled from two to three independent experiments. Statistical analysis was performed using two-tailed Student’s t-tests. ****P < 0.0001.

Given that T cell–derived IFN-γ promotes Th1-Treg differentiation, we next examined whether Treg cells themselves produce IFN-γ. To address this, we first re-analyzed RNA-seq datasets of YFP^+^ and YFP^−^ Treg cells isolated from the spleens of Foxp3-Cre/Tbx21-Flp/VeDTR(LF) mice (16). Differential gene expression analysis revealed that the Th1-like (YFP^+^) Treg subset expressed higher levels of *Ifng* and other Th1-associated genes, such as *Cxcr3* and *Tbx21*, compared with conventional (YFP^−^) Tregs (**Figure 3A**). A similar difference was observed after *in vitro* stimulation with anti-CD3ϵ and anti-CD28 antibodies in the presence of IL-2 (**Figure 3B**).

To confirm IFN-γ production at the protein level, tumor-infiltrating CD4^+^ T cells from MC38-bearing WT mice were stimulated *ex vivo* with PMA and ionomycin. Flow cytometric analysis demonstrated a distinct population of Foxp3^+^ CD4^+^ T cells that produced IFN-γ (**Figure 3C**). Notably, the frequency of IFN-γ-producing Tregs was significantly higher in the tumor than in the spleen. Together, these results indicate that Treg cells themselves are a source of IFN-γ within the tumor microenvironment. We further examined whether the frequency of IFN-γ-producing Tregs changes during tumor progression. Time-course analysis revealed that the proportion of IFN-γ^+^ Tregs was highest during the early stage of tumor development and gradually decreased as the tumor progressed to the mid and late stages (**Supplementary Figure 3**).

### Treg-derived IFN-γ reinforces Th1-Treg differentiation *in vitro*

**Figure 4:**
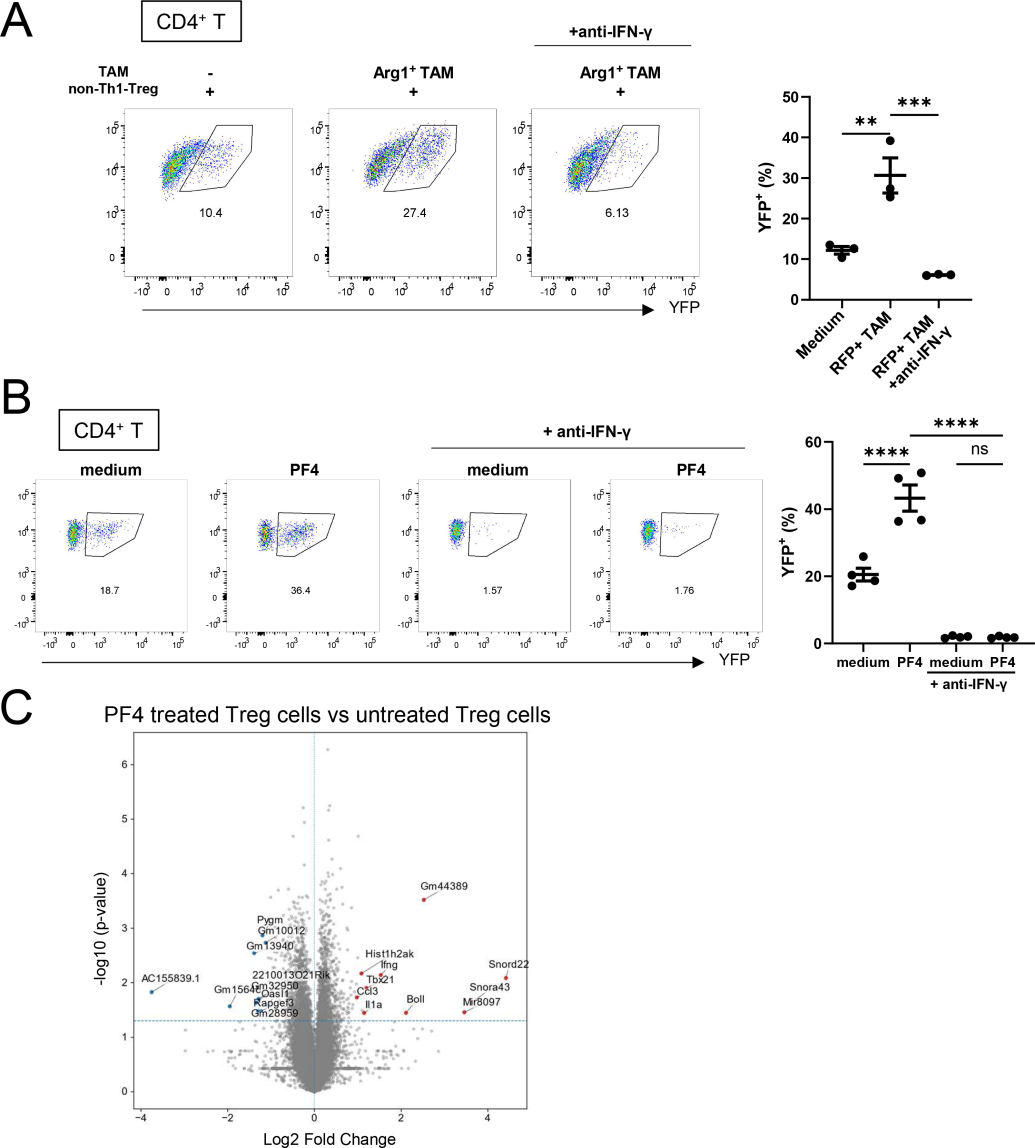
Treg-derived IFN-γ reinforces Th1-Treg differentiation *in vitro.* **(A)** Flow cytometry analysis of YFP^–^ CD4^+^ CD25^+^ cells that were derived from spleens of B16F10-bearing Foxp3-Cre/Tbx21-Flp/VeDTR(LF) mice and indirectly cocultured with RFP^+^ (Arg1^+^) TAMs from MC38-bearing Arg1-RFP mice by using transwell plates in the presence or absence of anti–IFN-γ monoclonal antibody. Gates show YFP^+^ cells. Representative FACS plots and the percentages of YFP^+^ cells are pooled from three independent experiments. **(B)** Flow cytometry analysis of YFP^–^ CD4^+^ CD25^+^ cells that were derived from spleens of B16F10-bearing Foxp3-Cre/Tbx21-Flp/VeDTR(LF) mice and subsequently stimulated with rPF4 in the presence or absence of anti–IFN-γ monoclonal antibody. Gates show YFP^+^ cells. Representative FACS plots and the percentages of YFP^+^ cells pooled from four independent experiments. **(C)** Volcano plots from bulk RNAseq analysis showing differential expression of genes in PF4 treated YFP^–^ CD4^+^ CD25^+^ from spleens of B16F10-bearing Foxp3-Cre/Tbx21-Flp/VeDTR(LF) mice. Data are mean ± SEM and pooled from two to three independent experiments. Statistical analysis was performed using one-way ANOVA followed by Dunnett’s multiple comparisons test. **P < 0.01; ***P < 0.001; ****P < 0.0001; ns, nonsignificant.

We next tested whether Treg-derived IFN-γ can promote the polarization of other Treg cells into Th1-Treg cells. Using a previously established transwell-based co-culture system (17), YFP^−^ Foxp3^+^ Treg cells from B16F10-bearing Foxp3-Cre/Tbx21-Flp/VeDTR (LF) mice were cultured with RFP^+^ (Arg1^+^) TAMs isolated from MC38 tumors of Arg1-RFP reporter mice. As previously reported (17), this co-culture significantly increased the proportion of YFP^+^ Foxp3^+^ Th1-Treg cells. Importantly, addition of an anti–IFN-γ neutralizing antibody substantially reduced this effect, indicating the critical role of IFN-γ in TAM-mediated Treg polarization (**Figure 4A**). Similarly, direct stimulation of Treg cells with PF4 promoted Th1-Treg differentiation, whereas concurrent blockade of IFN-γ abrogated this effect (**Figure 4B**). Transcriptome analysis further revealed that PF4 stimulation enhanced *Ifng* expression in Treg cells compared with untreated controls (**Figure 4C**). These results indicate that TAM-derived PF4 promotes Treg differentiation into Th1-Treg cells by amplifying IFN-γ expression, and that Treg-derived IFN-γ might be involved in this process.

### Treg-derived IFN-γ contributes to Th1-Treg formation *in vitro* and *in vivo*

**Figure 5:**
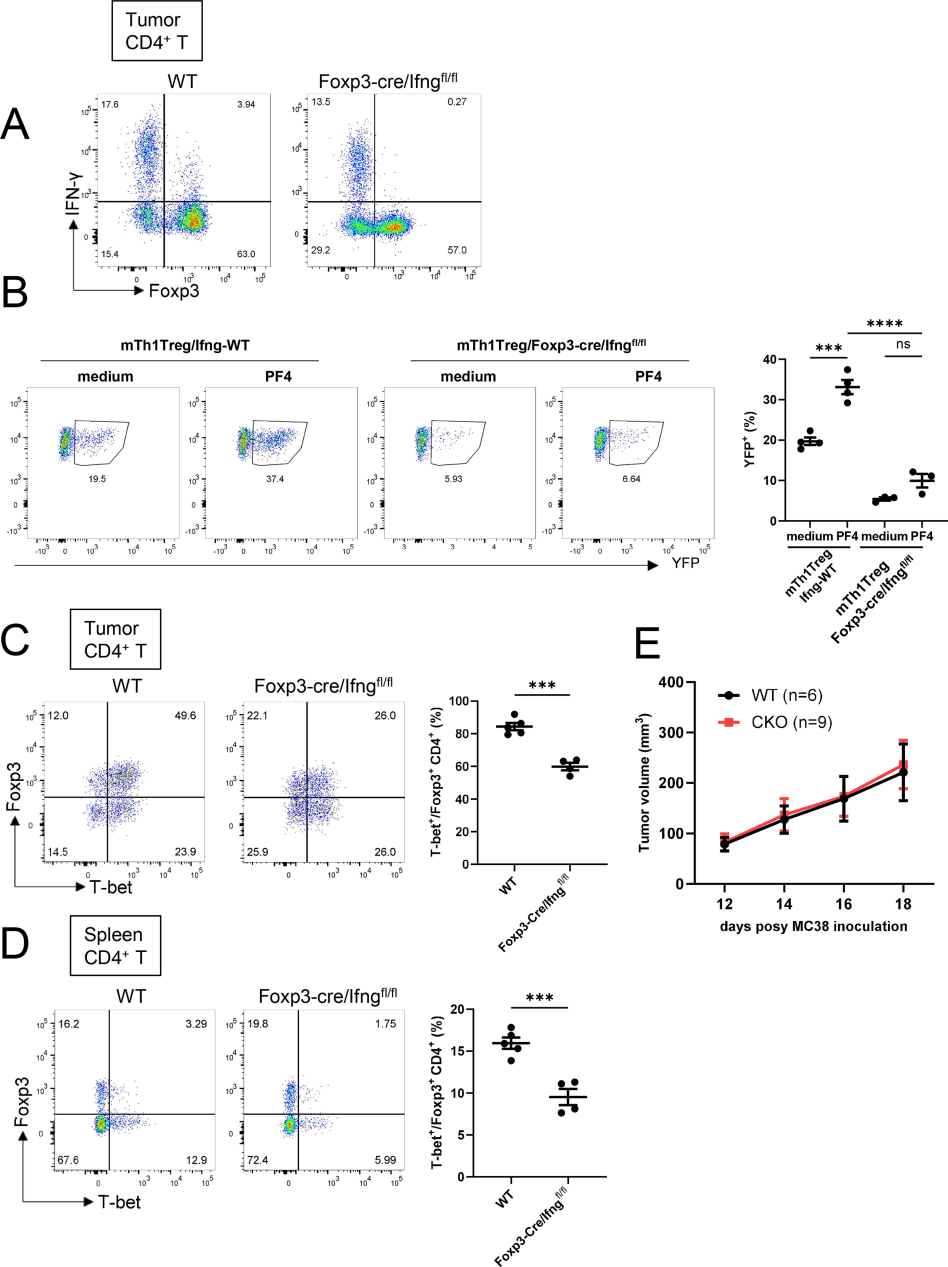
Treg-derived IFN-γ contributes to Th1-Treg formation *in vitro* and *in vivo.* **(A)** Flow cytometry analysis of Foxp3 and IFN-γ expression in tumor-infiltrating CD4^+^ T cells. CD4^+^ T cells were isolated from tumors of MC38-bearing WT mice or Foxp3-Cre/Ifng^fl/fl^ mice and stimulated *ex vivo* with PMA and ionomycin. Representative FACS plots showing Foxp3^+^ and IFN-γ^+^ populations. **(B)** Flow cytometry of YFP^–^ CD4^+^ CD25^+^ cells that were derived from spleens of B16F10-bearing Foxp3-Cre/Tbx21-Flp/VeDTR(LF) mice or and subsequently stimulated with recombinant PF4 protein (rPF4). Gates show YFP^+^ cells. Representative FACS plots and the percentages of YFP^+^ cells are pooled from three independent experiments. **(C, D)** Flow cytometry analysis of CD4^+^ T cells derived from tumors **(C)** or spleens **(D)** of WT or Foxp3-Cre/Ifng^fl/fl^ mice subcutaneously implanted with MC38 cells. Left, representative FACS plots; right, frequencies of T-bet^+^ cells among Foxp3^+^CD4^+^ T cells (n = 4 mice per group). **(E)** Growth of s.c. implanted MC38 tumors in WT or CD4-Cre/Ifng^fl/fl^ mice (indicated numbers of mice per group). Data are mean ± SEM and pooled from two to three independent experiments. Statistical analysis was performed using one-way ANOVA followed by Dunnett’s multiple comparisons test **(B)** and two-tailed Student’s t-tests **(C, D)**. *P < 0.05; ***P < 0.001; ****P < 0.0001; ns, non significant.

To directly test the role of Treg-derived IFN-γ, we generated Foxp3-Cre/Ifng^fl/fl^ mice, in which the *Ifng* gene is selectively deleted in Foxp3^+^ regulatory T cells. Phenotypic analysis revealed that Foxp3-Cre/Ifng^fl/fl^ mice were indistinguishable from control mice in terms of body weight and total cellularity in the spleen, lymph nodes, and brain (**Supplementary Figure 4**), indicating that Treg-specific *Ifng* deficiency does not affect gross development or basal immune homeostasis. Flow cytometric analysis confirmed the specific absence of IFN-γ expression in Foxp3^+^ Treg cells isolated from these mice (**Figure 5A**). When Treg cells isolated from Foxp3-Cre/Ifng^fl/fl^ mice were stimulated with PF4 *in vitro*, they failed to undergo polarization into Th1-Treg cells (**Figure 5B**). Furthermore, in tumor-bearing Foxp3-Cre/Ifng^fl/fl^ mice, the frequency of Th1-Treg cells was significantly reduced not only in the tumor microenvironment but also in the spleen compared with WT mice (**Figures 5C, D**). In terms of the absolute numbers of Th1-Tregs, we observed a downward trend in tumors, although it did not reach statistical significance, while the numbers in the spleen were comparable between the two groups (**Supplementary Figure 5A**).

We further examined the functional impact of this cellular phenotype on tumor progression. We found that tumor volume in Foxp3-Cre/Ifng^fl/fl^ mice was comparable to those in WT mice (**Figure 5E**). This raises the possibility that Treg-derived IFN-γ promotes Th1-Treg differentiation while also contributing to IFN-γ–dependent antitumor activity.

Furthermore, to evaluate the immunosuppressive potential of the remaining Tregs, we assessed the expression of key molecules involved in Treg suppression, such as CD25, CTLA-4, and TIGIT (22, 23). We found no significant differences in the expression levels of these markers between WT and Foxp3-Cre/Ifng^fl/fl^ mice (**Supplementary Figure 5B**), indicating that the expression of key suppressive markers in Tregs is largely preserved despite impaired Th1-Treg differentiation.

### Treg-derived IFN-γ contributes to Th1-Treg differentiation in autoimmune inflammation

**Figure 6:**
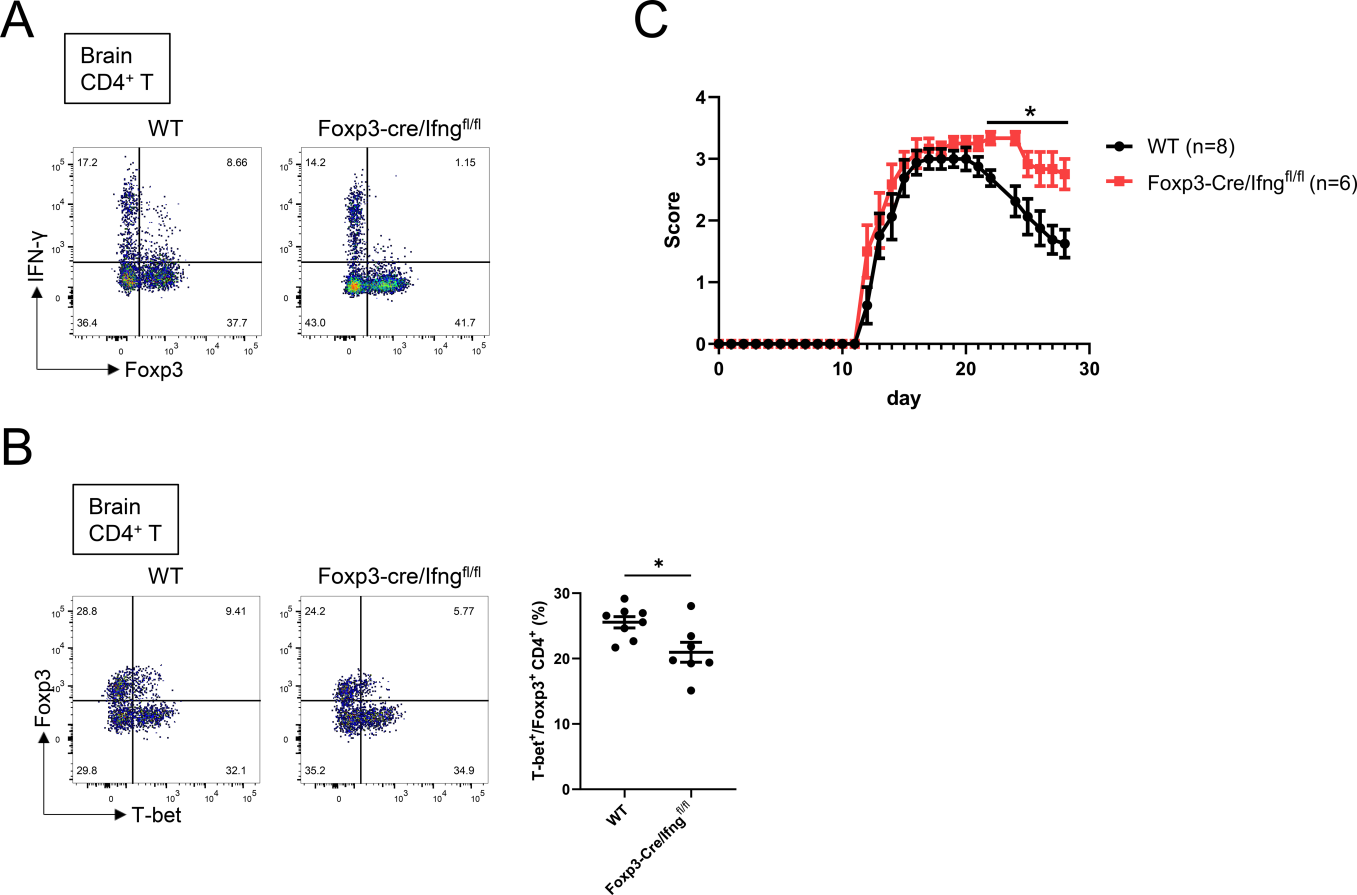
Treg-derived IFN-γ contributes to Th1-Treg differentiation in autoimmune inflammation. **(A)** Flow cytometry analysis of Foxp3 and IFN-γ expression in brain-infiltrating CD4^+^ T cells at the peak of EAE. CD4^+^ T cells were isolated from the brains of WT or Foxp3-Cre/Ifng^fl/fl^ mice at the peak disease stage and stimulated *ex vivo* with PMA and ionomycin. Representative FACS plots showing Foxp3^+^ and IFN-γ^+^ populations. **(B)** Flow cytometry analysis of CD4^+^ T cells derived from the brains of WT or Foxp3-Cre/Ifng^fl/fl^ mice at the peak of EAE. (Left) Representative FACS plots and (right) the percentages of T-bet^+^ cells among Foxp3^+^CD4^+^ T cells in the brains (n = 7–8 mice per group). **(C)** Clinical scores of EAE in WT and Foxp3-Cre/Ifng^fl/fl^ mice (indicated numbers of mice per group). Data are mean ± SEM and pooled from two to three independent experiments. Statistical analysis was performed using two-tailed Student’s t-tests. *P < 0.05.

It remains unclear whether Treg-derived IFN-γ also contributes to Th1-Treg differentiation under non-tumor inflammatory conditions. Since we have shown that Th1-Treg cells play an important role in experimental autoimmune encephalomyelitis (EAE) (19), we finally examined the role of Treg-derived IFN-γ in Th1-Treg differentiation during EAE. Following EAE induction, we analyzed Foxp3 and IFN-γ expression in brain-infiltrating T cells and found that a part of Tregs expressed IFN-γ (**Figure 6A**). Consistently, Foxp3-Cre/Ifng^fl/fl^ mice exhibited a loss of IFN-γ expression in Foxp3^+^ Tregs within the inflamed brain (**Figure 6A**). Furthermore, analysis of Foxp3^+^ T cells in the brains of Foxp3-Cre/Ifng^fl/fl^ mice revealed a significant reduction in the proportion of T-bet^+^Foxp3^+^ Th1-Treg cells compared with wild-type controls (**Figure 6B**). To evaluate the functional consequence of this impaired Th1-Treg differentiation, we monitored the clinical severity of EAE. We found that Foxp3-Cre/Ifng^fl/fl^ mice exhibited significantly higher EAE clinical scores compared with wild-type controls (**Figure 6C**). Taken together, these results indicate that Treg-derived IFN-γ promotes Th1-Treg differentiation and is required for the suppression of autoimmune inflammation.

## Discussion

In this study, we identified Treg-derived IFN-γ as a factor that reinforces the differentiation and maintenance of Th1-Treg cells both in tumors and during autoimmune inflammation. Although IFN-γ is classically recognized as a pro-inflammatory cytokine that promotes Th1 effector differentiation and antitumor immunity, our findings uncover a paradoxical, immunoregulatory role of IFN-γ signaling—namely, its capacity to sustain a regulatory subset within the TME. Specifically, Treg-derived IFN-γ acts in an autocrine manner to stabilize the Th1-Treg phenotype, and this process is further amplified by TAM–derived PF4, forming a self-reinforcing circuit that promotes immune suppression within tumors. This mechanism reveals the dual nature of IFN-γ, which acts as an inflammatory cytokine in normal immunity but as an immunosuppressive mediator within the tumor environment.

Regulatory T cells are increasingly recognized as a plastic population that adapts to local cytokine and metabolic signals (6, 11). Previous studies have shown that IFN-γ receptor signaling induces T-bet expression in Tregs, thereby enhancing their stability under Th1-skewed conditions (12, 24, 25). Here, we extend this concept by identifying Tregs as one of the sources of IFN-γ that support the differentiation of Th1-Tregs. While global loss of IFN-γ or its T cell–specific deletion produces a stronger phenotype than Treg-specific deletion, the latter still reduces the abundance of Th1-Treg cells, suggesting that IFN-γ derived from Tregs contributes, at least in part, to the differentiation of Th1-Treg cells, in cooperation with IFN-γ from other T cell subsets such as Th1 and CD8^+^ T cells. Notably, the reduction of Th1-Treg frequency in Foxp3-Cre/Ifng^fl/fl^ mice was observed not only in tumor but also in the spleen, suggesting that Treg-derived IFN-γ contributes to the maintenance of Th1-Treg cells both locally and systemically. This finding suggests that, while the effect of PF4 is largely confined to the tumor microenvironment (17), IFN-γ exerts broader, systemic effects that contribute to the maintenance of Th1-Treg cells beyond the tumor site.

An important observation in our study is that, despite the reduced frequency of suppressive Th1-Treg cells in Foxp3-Cre/Ifng^fl/fl^ mice (**Figure 5C**), overall tumor growth was not significantly altered (**Figure 5E**), and this finding requires careful interpretation. We believe this result reflects the dual and context-dependent roles of IFN-γ within the tumor microenvironment. On the one hand, IFN-γ promotes the differentiation and maintenance of highly suppressive Th1-Treg cells, which restrain antitumor immunity. On the other hand, IFN-γ itself is an critical antitumor cytokine that enhances cytotoxic CD8+ T cells and NK cells. Thus, in Foxp3-Cre/Ifng^fl/fl^ mice, selective loss of IFN-γ from Treg cells reduces the Th1-Treg population but may simultaneously attenuate Treg-derived IFN-γ–dependent antitumor effector functions, resulting in a net neutral effect on tumor growth. In contrast, global or broader T cell–specific deletion of *Ifng* (Ifng^^−^/^−^^ and CD4-Cre/Ifng^fl/fl^ mice) removes IFN-γ from effector compartments as well and leads to accelerated tumor growth, underscoring the dominant antitumor role of IFN-γ. Importantly, this lack of a tumor growth phenotype does not indicate that Th1-Treg cells are functionally irrelevant. Rather, it reflects the dual and counterbalancing functions of IFN-γ in the tumor microenvironment, where IFN-γ simultaneously supports immunosuppressive Th1-Treg differentiation and antitumor effector responses. As a result, selective loss of IFN-γ in Treg cells reduces the Th1-Treg compartment but may be offset by concurrent changes in IFN-γ–dependent effector pathways, leading to no overt alteration in tumor growth.

We also found that the expression of canonical Treg-associated suppressive molecules, including CD25, CTLA-4, and TIGIT, was largely unchanged in Foxp3-Cre/Ifng^fl/fl^ mice. This observation suggests that deletion of *Ifng* in Tregs does not broadly alter the expression of key suppressive molecules. Instead, the primary effect of Treg-specific IFN-γ deficiency appears to be a reduction in the frequency of the Th1-Treg subset, while the Treg suppressive phenotype is preserved.

The frequency of T-bet^+^Foxp3^+^ Tregs was higher than that of IFN-γ-producing Tregs, indicating that not all Th1-Tregs actively produce IFN-γ. This is consistent with the notion that T-bet expression reflects lineage adaptation and functional potential rather than ongoing cytokine secretion. IFN-γ production was assessed at the protein level following ex vivo stimulation with PMA and ionomycin, which captures only the subset of Tregs that are competent to produce the cytokine under these conditions. Taken together, these observations suggest that the Th1-Treg population might be heterogeneous, consisting of both IFN-γ-producing and non-producing cells.

Mechanistically, our results reveal that TAM-derived PF4 is functionally linked to Treg-derived IFN-γ. PF4 stimulation induced *Ifng* transcription in Tregs, which in turn promoted further Th1-Treg differentiation through an IFN-γ-dependent mechanism. This macrophage–Treg interplay illustrates how the TME repurposes inflammatory signaling pathways to reinforce immune suppression.

Recent studies have described a distinct ontogenic route in which IFN-γ–producing Th1 cells acquire Foxp3 and T-bet expression through TGF-β signaling within tumors, giving rise to CD39^+^ pTregs with potent suppressive activity (26). While this study focused on the origin of FOXP3^+^T-bet^+^ regulatory T cells, our results reveal another regulatory mechanism operating within existing Treg cells, showing how PF4-induced Treg cell-intrinsic IFN-γ signaling maintains and expands the established Th1-Treg phenotype.

Although IFN-γ-producing Treg cells have been reported in various pathological conditions (27–29), their functional significance in Th1-Treg differentiation has remained unclear. Previous studies showed that Foxp3^+^ Treg cells can co-produce IFN-γ and IL-10 under Th1-skewed inflammatory conditions such as viral encephalitis, where they suppress the proliferation of epitope-matched effector CD4^+^ T cells while retaining their regulatory activity (30). Other studies demonstrated that IFN-γ signaling promotes the formation of Th1-like Treg cells capable of functioning in IFN-γ–rich environments (11, 31, 32). These findings indicate that IFN-γ plays an important role in supporting adaptation to Th1-dominant inflammation by maintaining the suppressive capacity of Treg cells. Our current study reveals that IFN-γ produced by Treg cells is not simply a byproduct of activation but plays a substantial role to maintain the Th1-Treg lineage.

Beyond cancer, our experiments in EAE demonstrate that Treg-derived IFN-γ likewise promotes Th1-Treg differentiation during autoimmune inflammation, consistent with previous reports showing that Th1-Tregs limit tissue damage by restraining pathogenic Th1 cells (11, 19, 33, 34). Thus, IFN-γ–producing Tregs contribute to immune suppression in distinct contexts—limiting excessive inflammation and tissue damage during autoimmunity, while promoting tumor-associated immune suppression in cancer. Consistent with these findings, Foxp3-Cre/Ifng^fl/fl^ mice showed increased EAE clinical scores, indicating that loss of IFN-γ specifically in Tregs is associated with exacerbated autoimmune disease.

Together, Treg cells produce IFN-γ, which contributes to the differentiation of Th1-Treg cells. Arg1^+^ TAM–derived PF4 enhances this process by inducing IFN-γ expression in Treg cells, thereby facilitating the accumulation of Th1-Treg cells within the TME and contributing to tumor-associated immune suppression.

## Materials and methods

### Cell lines

MC38 mouse colon carcinoma cells and B16F10 mouse melanoma cells were as described previously (16). MC38 cells were cultured in RPMI1640 (Nacalai Tesque) with 10% heat-inactivated FBS (Gibco), 100 U/ml penicillin, and 0.1 mg/ml streptomycin (Nacalai Tesque). B16F10 cells were cultured in DMEM (Nacalai Tesque) with 10% heat-inactivated FBS (Gibco), 100 U/ml penicillin, and 0.1 mg/ml streptomycin (Nacalai Tesque). Cells were maintained in culture at 37 °C and 5% CO_2_.

### Mice

C57BL/6NCrSlc (C57BL/6N; CD45.2) mice were purchased from Japan SLC, Inc. Foxp3-Cre mice, Foxp3-Cre/Tbx21-Flp/VeDTR(LF) mice were described as previously (16). CD4-Cre mice, *Ifng*^−/−^ mice and *Ifng*^fl/fl^ mice were described previously (35). Mice were euthanized by carbon dioxide (CO_2_) inhalation delivered at a flow rate of 30–70% of the chamber volume per minute, unless otherwise specified. All animal experiments were conducted with the approval of the Animal Research Committee of Research Institute for Microbial Diseases in Osaka University.

### Reagents

PerCP/Cyanine5.5 anti-mouse CD45.2 Antibody (Cat#109828), PerCP/Cyanine5.5 anti-mouse CD25 Antibody (Cat#102030), PE/Cyanine7 anti-mouse CD4 Antibody (Cat#100422), Brilliant Violet 421 anti-mouse CD8a Antibody (Cat#100738), Brilliant Violet 421 anti-mouse/human CD45R/B220 Antibody (Cat#103240), Brilliant Violet 421 anti-mouse CD11c Antibody (Cat#117330), PE anti-mouse IFN-γ Antibody (Cat#505808), Brilliant Violet 421 anti-mouse/human CD11b Antibody (Cat#101236), Alexa Fluor^®^ 647 anti-mouse FOXP3 Antibody (Cat#126408), PE anti-mouse CD25 Antibody (Cat#102008), PE anti-mouse CD152 Antibody (Cat#106306), PE anti-mouse TIGIT (Vstm3) Antibody (Cat#142103) and recombinant mouse CXCL4/PF4 (Cat#590208) were purchased from BioLegend. PE Mouse anti-T-Bet (Cat#561265) was purchased from BD Pharmingen. InVivoMAb anti-mouse IFNγ (Cat#BE0055) was purchased from Bio X cell. DAPI (Cat#11034-56) was purchased from Nacalai Tesque.

### Murine tumor model

Tumor cells were injected subcutaneously into 7-11-week-old sex-matched mice. 1.0 × 10^6^ MC38 cells or 3.0 × 10^5^ B16F10 cells in phosphate-buffered saline (PBS) were injected subcutaneously to the back of mice. Tumor volume was measured using a digital caliper and calculated by the following formula: (short diameter)^2^ × long diameter × 0.52. When the calculated tumor volume was larger than 2500 mm^3^, mice were euthanized within 24 hours. Without specific indication, tumor-bearing mice were analyzed at 15–21 days after tumor inoculation.

### Cell preparation from mice

Spleens were dissociated in PBS by gently mashing through a 70-μm cell strainer using the plunger of a syringe. The cell suspension was centrifuged for 5 min at 2000 rpm, resuspended in ACK lysis buffer, and incubated for 2 min at room temperature to remove red blood cells. After lysis, cells were washed with PBS and passed through a 40-μm cell strainer.

Tumor tissues were processed using the Mouse Tumor Dissociation Kit (Miltenyi, Cat# 130-096-730) and the gentleMACS Octo Dissociator with Heaters (Miltenyi) according to the manufacturer’s protocol. Following enzymatic digestion, the cell suspension was treated with ACK buffer to lyse red blood cells, and dead cells were removed using the Dead Cell Removal Kit (Miltenyi, Cat# 130-090-101). The resulting single-cell suspensions were resuspended in the appropriate buffer for downstream experiments.

To prepare cells from the brain, mice were euthanized with 5% isoflurane inhalation and transcardially perfused with 10 mL of HBSS. Brains were gently dissociated in HBSS by mashing through a 70-μm cell strainer using the plunger of a syringe. The cell suspension was centrifuged for 5 min at 2000 rpm and treated with ACK lysis buffer to remove red blood cells. Cells were then resuspended in 40% Percoll (Sigma-Aldrich) prepared in HBSS and centrifuged for 20 min at 2,380 × g to eliminate myelin and floating debris. The resulting cell pellet was washed once with HBSS and used for subsequent analyses.

### Flow cytometry and cell sorting

For surface staining, cells were blocked with anti-CD16/32 (Biolegend) for 15 min on ice. Cells were stained with antibodies for 15 min on ice in the presence of anti-CD16/32. For intranuclear staining, Foxp3/Transcription Factor Staining Buffer Set (eBioscience, Cat#00-5523-00) was used following the manufacturer’s instruction. FACS Aria III (BD Biosciences) was used for data acquisition. The acquired data were analyzed using FlowJo (BD Biosciences).

### RNA-seq

For RNA-seq analysis, total RNA was extracted from cells with a miRNeasy Mini kit (Qiagen, Cat#217004) following the manufacturer’s instruction. Full-length cDNA was generated using SMART-Seq HT Kit (Takara Bio, Cat#634455) following the manufacturer’s instruction. An Illumina library was prepared using a Nextera DNA Library Preparation Kit (Illumina, Cat#FC-131-1096) following SMARTer kit instructions. Sequencing was performed on an Illumina NovaSeq 6000 sequencer (Illumina) in the 101-base single-end mode. Sequenced reads were mapped to the mouse reference genome sequences (mm10) using TopHat v2.0.13 in combination with Bowtie2 ver. 2.2.3 and SAMtools ver. 0.1.19. The fragments per kilobase of exon per million mapped fragments (FPKMs) was calculated using Cufflinks version 2.2.1.

### EAE induction

Experimental autoimmune encephalomyelitis (EAE) was induced in age- and sex-matched mice (9–16 weeks old) by subcutaneous immunization at two sites on the back with 200 μL of an emulsion containing 100 μL PBS with 2 mg/mL MOG35–55 peptide (ProSpec) and 100 μL complete Freund’s adjuvant (CFA) supplemented with 5 mg/mL heat-killed Mycobacterium tuberculosis H37RA (Chondrex). Mice received intraperitoneal injections of 500 ng pertussis toxin (Millipore) immediately after immunization and again 2 days later. Clinical signs of EAE were monitored daily and scored according to the Mouse EAE Scoring Guide provided by Hooke Laboratories. 

Clinical scores were defined as follows:0, no clinical signs;0.5, tip of the tail is limp;1, limp tail;1.5, limp tail and hind limb weakness;2, limp tail and weakness of the hind limbs;2.5, limp tail and dragging of the hind limbs;3, limp tail and complete paralysis of the hind limbs;4, limp tail, complete hind limb paralysis and partial forelimb paralysis;4.5, complete hind limb and partial forelimb paralysis with minimal movement in the cage;5, moribund.

### Statistical analysis

All quantitative data are shown as mean ± standard error of mean (SEM) and were analyzed using GraphPad Software (Prism v10.0.3). Two-tailed Student’s *t* test was used to compare two datasets. One-way or two-way analysis of variance (ANOVA) followed by Tukey’s or Dunnett’s multiple comparisons test was used to detect the differences in the results between groups. P values < 0.05 were considered statistically significant.

## Data Availability

The data presented in the study are deposited in the Gene Expression Omnibus (GEO) repository under accession number GSE327115. The study also includes re-analysis of previously published data (GSE197647), which are publicly available in GEO.

## Supplementary material

Supplementary Figure 1 Th1-Treg cell numbers in tumors and spleen of WT and Ifng^−^/^−^ mice. **(A)** Numbers of Th1-Tregs per mg of tumor or spleen tissue in WT and Ifng^-/-^ mice s.c. implanted with MC38 cells (n = 4 per group). Data are mean ± SEM and pooled from two to three independent experiments. Statistical analysis was performed using two-tailed Student’s t-tests. **, P < 0.01; ns, nonsignificant.

Supplementary Figure 2 Th1-Treg cell numbers in tumors and spleen of WT and CD4-Cre/Ifng^fl/fl^ mice. **(A)** Numbers of Th1-Tregs per mg of tumor or spleen tissue in WT and CD4-Cre/Ifng^fl/fl^ mice s.c. implanted with MC38 cells (n = 5 per group). Data are mean ± SEM and pooled from two to three independent experiments. Statistical analysis was performed using two-tailed Student’s t-tests. *, P < 0.05; **, P < 0.01.

Supplementary Figure 3 IFN-γ production in Tregs at different stages of tumor growth. Flow cytometry analysis of Foxp3 and IFN-γ expression in tumor-infiltrating CD4^+^ T cells isolated from MC38-bearing WT mice at early (10 days after tumor inoculation), mid (20 days after tumor inoculation), and late stages (40 days after tumor inoculation) and stimulated *ex vivo* with PMA and ionomycin. Representative FACS plots and percentages of Foxp3^+^IFN-γ^+^ cells among tumor-infiltrating CD4^+^ T cells are shown. Data are mean ± SEM and pooled from two to three independent experiments. Statistical analysis was performed using two-tailed Student’s t-tests. **, P < 0.01; ***, P < 0.001; ns, nonsignificant.

Supplementary Figure 4 Treg-specific deletion of *Ifng* does not affect body weight or immune cellularity. **(A, B)** Body weight **(A)** and total cell numbers **(B)** in the spleen, lymph nodes, and brain were compared between WT control and Foxp3-Cre/Ifng^fl/fl^ mice (n = 3–5 per group). Data are mean ± SEM and pooled from two to three independent experiments. Statistical analysis was performed using two-tailed Student’s t-tests. ns, nonsignificant.

Supplementary Figure 5 Analysis of Treg numbers and suppressive marker expression in Foxp3-Cre/Ifng^fl/fl^ mice. **(A)** Numbers of Th1-Tregs per mg of tumor or spleen tissue in WT and Foxp3-Cre/Ifng^fl/fl^ mice s.c. implanted with MC38 cells (n = 5 per group). **(B)** Expression of TIGIT, CD25, and CTLA-4 in tumor infiltrating Tregs from WT and Foxp3-Cre/Ifng^fl/fl^ mice. Representative histogram overlays and measurements of mean fluorescence intensity (MFI). Data are mean ± SEM and pooled from two to three independent experiments. Statistical analysis was performed using two-tailed Student’s t-tests. ns, nonsignificant.
