# Supplementary material for: Treg-derived IFN-γ supports the differentiation of Th1-Treg in tumor immunity and autoimmunity
Source: Front Immunol. 2026 May 12;17:1756194. doi: 10.3389/fimmu.2026.1756194 (PMC13201394; doi:10.3389/fimmu.2026.1756194)

Supplementary Figure 1

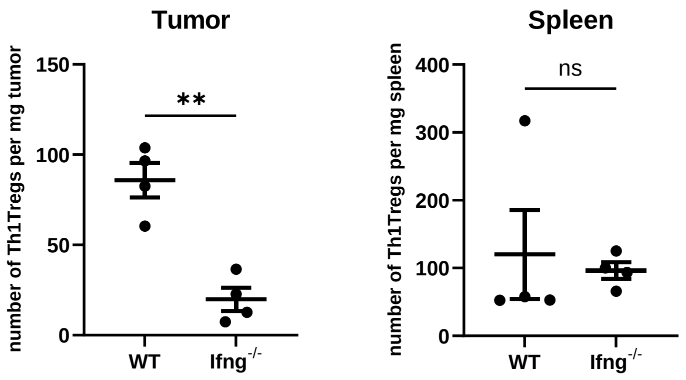

Supplementary Figure 2

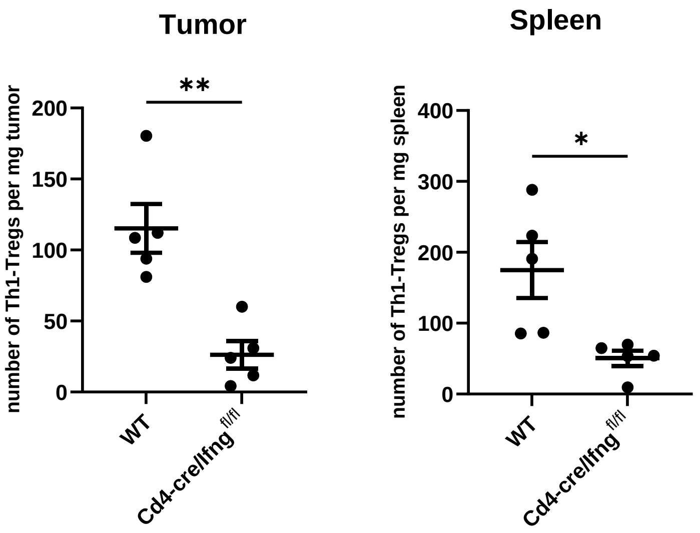

Supplementary Figure 3

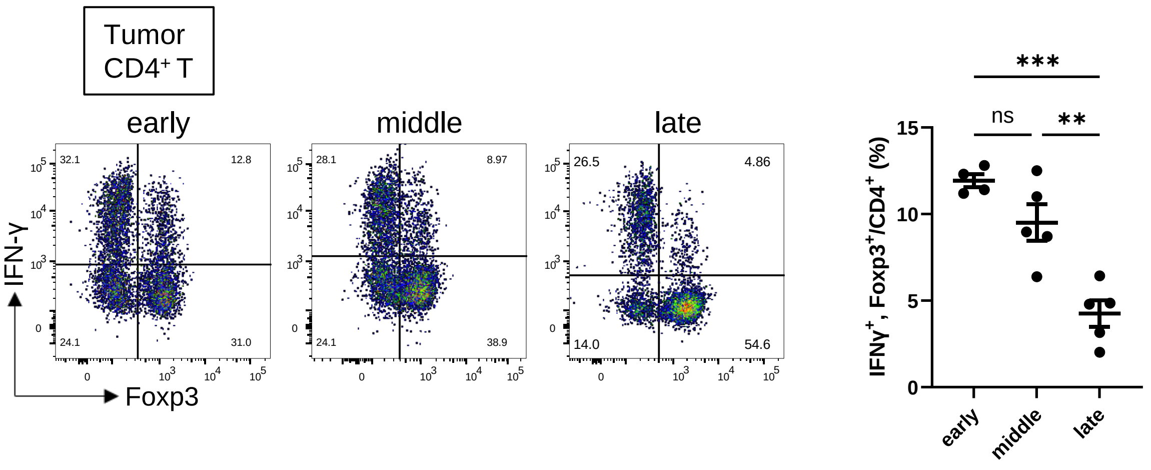

Supplementary Figure 4

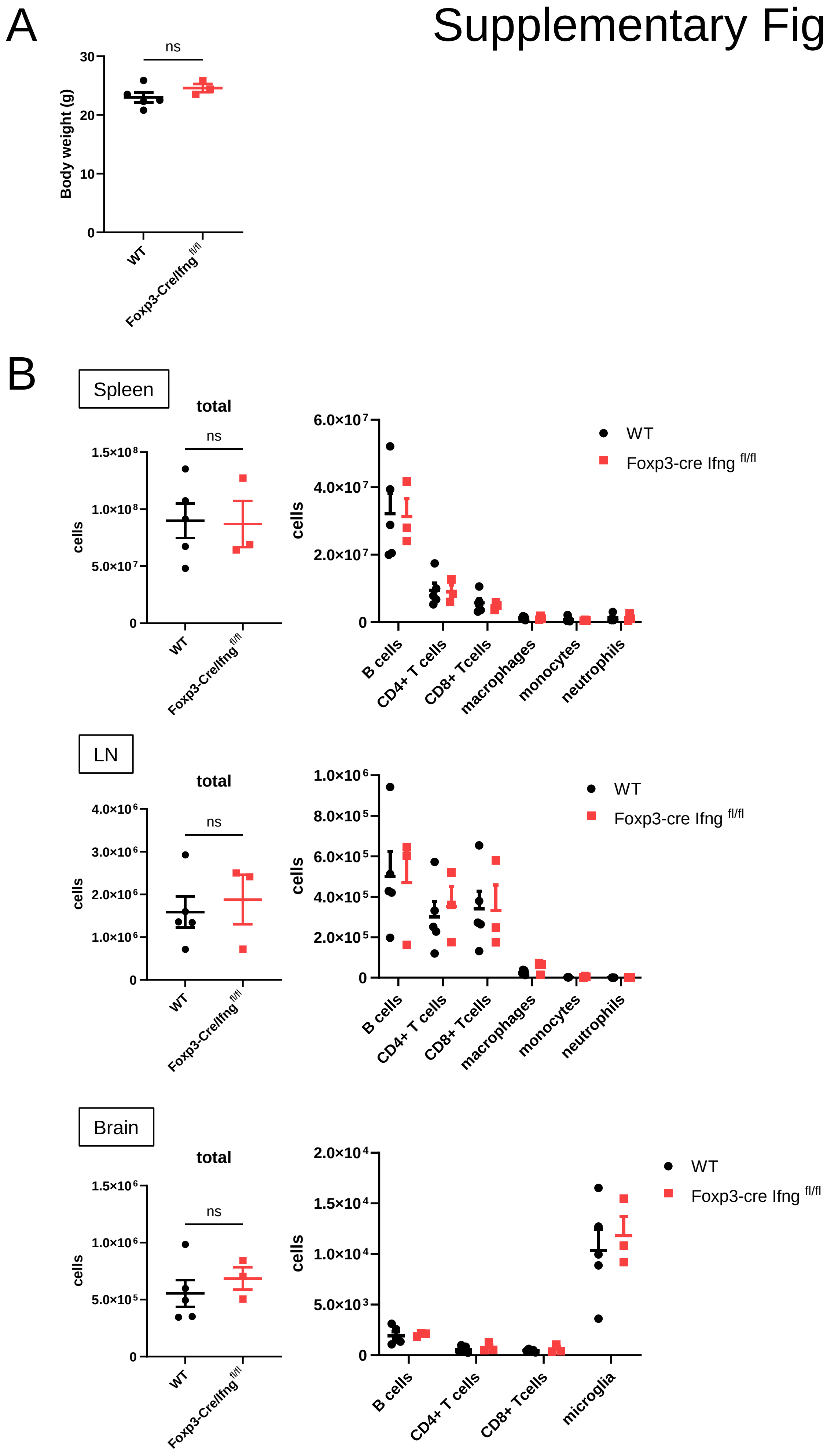

Supplementary Figure 5

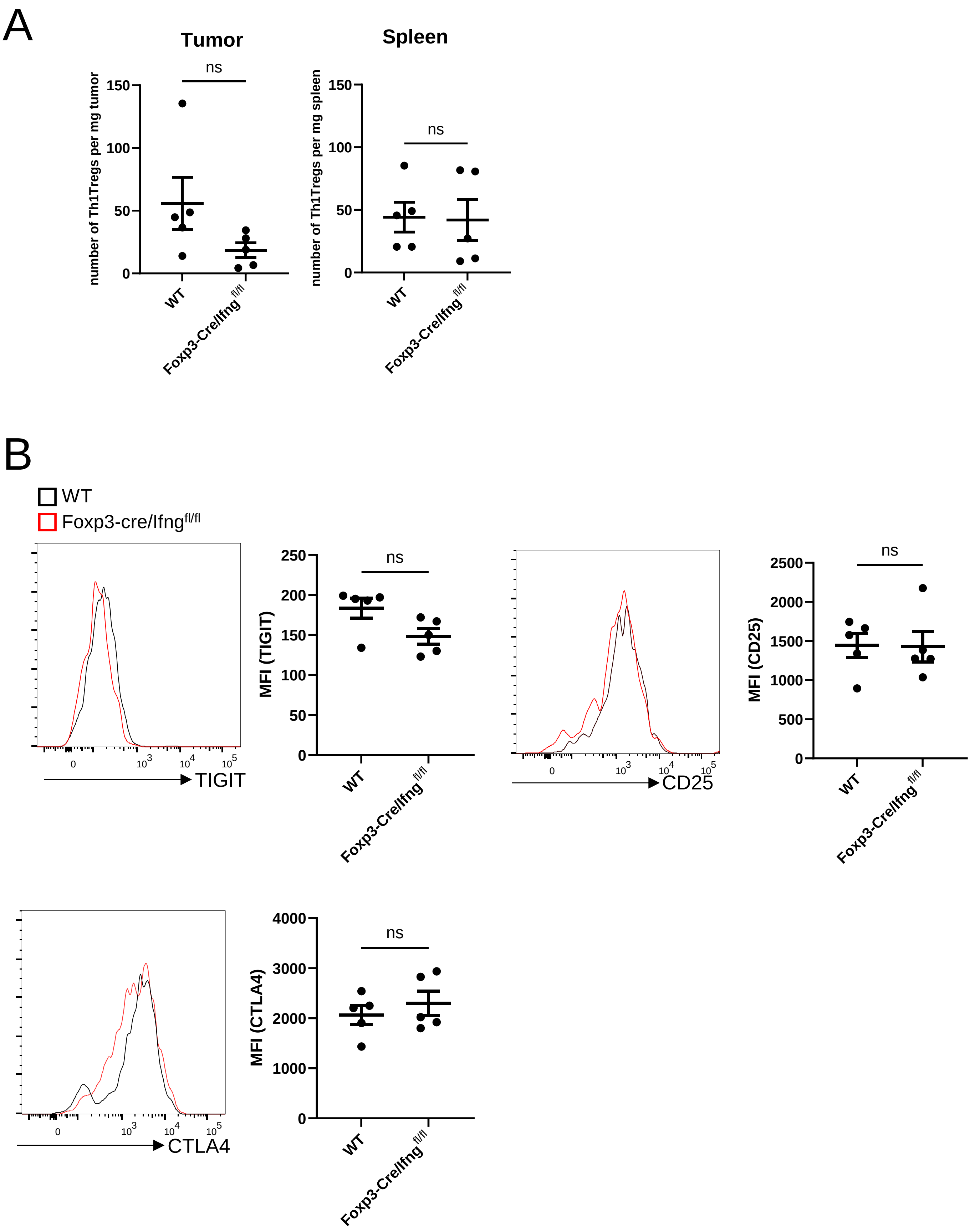

Supplement: Supplementary Figure 1 — Th1-Treg cell numbers in tumors and spleen of WT and Ifng−/− mice. (A) Numbers of Th1-Tregs per mg of tumor or spleen tissue in WT and Ifng-/- mice s.c. implanted with MC38 cells (n = 4 per group). Data are mean ± SEM and pooled from two to three independent experiments. Statistical analysis was performed using two-tailed Student’s t-tests. **, P < 0.01; ns, nonsignificant. [file Supplementaryfile1.pdf]
